# On a Method of Obtaining Homogeneous Light of Great Intensity

**Published:** 1861-07

**Authors:** 


ON A METHOD OF OBTAINING HOMOGENEOUS LIGHT OF GREAT INTENSITY.—As it is a desideratum in optical science to procure *perfectly* homogeneous light of sufficient brightness for many important experiments, I am glad to be able to communicate a method which, in a satisfactory manner, supplies that deficiency. It is only requisite to place a lump of common salt upon the wick of a spirit-lamp, and to direct a stream of oxygen gas from a blow-pipe upon the salt. The light emitted is quite homogeneous and of dazzling brightness. If, instead of common salt, we use the various salts of strontium, barytes, etc., we obtain the well-known colored flames which are characteristic of those substances with far more brilliancy than by any other method with which I am acquainted.—*Mr. H. F. Talbot in Chemical News.*

---

THE MICROSCOPE discovers the fact that the mould on bread and other provisions in damp, warm weather, is a dense forest in miniature, with buds, leaves, flowers, and fruit.

